# Progress in the Development of Anti-tissue Factor Pathway Inhibitors for Haemophilia Management

**DOI:** 10.3389/fmed.2021.670526

**Published:** 2021-05-05

**Authors:** Johnny N. Mahlangu

**Affiliations:** Haemophilia Comprehensive Care Centre, Charlotte Maxeke Johannesburg Academic Hospital, University of the Witwatersrand and National Health Laboratory Service, Johannesburg, South Africa

**Keywords:** haemophilia, tissue factor pathway inhibitor, monoclonal antibody, non-factor replacement therapy, marstacimab, concizumab, BAY 1093884, anti-TFPI

## Abstract

The unprecedented progress in addressing unmet needs in haemophilia care to date includes developing several novel therapies that rebalance haemostasis by restoring thrombin generation in patients with haemophilia A or B with and without inhibitors. These novel therapies are FVIII mimetics, antithrombin interference RNA therapy and several monoclonal antibodies directed against the tissue factor pathway inhibitor (anti-TFPI). In this review, we provide an update on the progress made in developing anti-TFPI therapie. Phase 1 data from the three anti-TFPI studies showed acceptable safety profiles, and currently, available phase 2 data are encouraging. While these data support these molecules' further development progression, there is uncertainty on several aspects of their evolution. Two of the three anti-TFPIs have shown drug-related thrombosis, with one study consequently terminated. None of the thrombotic events is predictable with current monitoring tools, and none correlate with known coagulation parameters. All three anti-TFPIs undergo target mediated drug disposition, which impacts the formulation of dosing regimen fo these therapies. They would require more frequent dosing than some of the extended half-life clotting factor products and antithrombin RNAi therapy. There is no assay to measure the TFPI as the physiological levels are very low, which makes monitoring the impact of the anti-TFPI a challenge. The anti-TFPIs have several advantages, including their bioavailability when administered subcutaneously, their stable pharmacokinetics and their ability to prevent bleeds in haemophilia A or B patients with and without inhibitors. Whether these advantages can be realized will depend on the outcome of the currently ongoing studies.

## Introduction

Haemophilia is a rare X-linked inherited bleeding disorder characterised by remarkable management progress in the past several decades. Advances in novel haemophilia therapy development have primarily been driven by the efforts to address the many currently unmet needs in haemophilia care, which include the high disease and treatment burden (1–3), the need for intravenous infusion of factor replacement therapy (4–8), the immunogenicity of factor replacement therapy (9–11), suboptimal prophylaxis due to poor compliance and adherence (12). The non-factor replacement therapies (NFRT) ware developed to address some of these unmet needs in haemophilia care.

The unprecedented progress in the development of NFRT in the last decade includes developing factor VIII (FVIII) mimetics, antithrombin RNA interference and several tissue factor pathway inhibitors (TFPI). Potential inhibitors of TFPI have included an aptamer and several monoclonal antibodies directed against this natural inhibitor of coagulation. While the anti-TFPI aptamer showed promise in the preclinical evaluations (13, 14), it resulted in increased bleeding at its highest dose when tried in humans with consequent termination of its clinical development program (15).

The aptamer termination disappointment was followed by a switch to the development of monoclonal antibodies, of which three with anti-TFPI activities have been engineered. These are the befovacimab, concizumab and marstacimab. All three anti-TFPI molecules target the Kunitz-2 (K2) domain of the TFPI, with the befovacimab also targeting the Kunitz 1 (K1) TFPI domain. Under physiological conditions, the TFPI molecule binds activated Factor X (FXa) via its K2 domain to form the TFPI-FXa complex which inhibits FXa. The TF-FVIIa complex then binds the TFPI-FXa through the TFPI K1 domain to form the TFPI-FXa-TF-FVIIa quartenary complex with resultant inhibition of FVIIa. Therefore, binding of TFPI to the K2 is a rate limiting steps in this haemostasis inhibition process making development of anti-TFPI therapies directed against the TFPI K2 domain an attractive target for anti-TFPI therapy development.

Studies on the evaluation of these novel molecules have been ongoing for the last 5 years. This review will summarise currently available clinical data on the development of anti-TFPI monoclonal antibodies. The pertinent characteristics of the anti-TFPI monoclonal antibodies and their progress in human studies are summarised in Table 1. The adverse events of special interest during the conduct of the anti-TFPI moleculare are summarized in Table 2.

**Table 1:** Characteristics and progress in the development of anti-TFPI monoclonal antibodies.

**Anti-TFPI monoclonal antibody (developer)**	**Monoclonal antibody features**	**TFPI Binding site**	**Phase 1 study identifier and progress**	**Phase 2 study identifier and progress**
Befovacimab (Bayer)	Human IgG2	K1 and K2 domains	NCT02571569/ NCT03481946 Completed both	NCT03597022 Terminated
Concizumab (NovoNordisk)	Humanised IgG4	K2 domain	NCT02490787 completed	NCT03196297Completed
Marstacimab (Pfizer)	Human IgG1	K2 domain	NCT02531815 completed	NCT03363321Completed
MG1113 (Greencross)	Humanised IgG4	K2 domain	NCT03855696 Ongoing	Not yet started

*TFPI, tissue factor pathway inhibitor; IgG, immunoglobulin isotype G; K1, Kunitz domain 1; K2, Kunitz domain 2*.

**Table 2:** Adverse events of special interest in phase 2 anti-TFPI clinical trials (19, 24, 28).

**Anti-TFPI antibody**	**Number of participants exposed (*n*)**	**Post exposure follow-up period (weeks)**	**Thrombosis deaths**	**Platelet/fibrinogen change**	**Allergy/anaphylaxis**	**Anti-drug antibodies**	**Neutralising antibodies**
Befovacimab	24 (8 in each 100 mg, 225 mg and 400 mg dose cohort)	2-47	3 thromboses (1 venous and 2 arterial) No death	None	Not reported- study terminated	Not reported study terminated	Not reported study terminated
Concizumab	53 (36 HA, 9 HAwI, 8 HBwI)	24	No thrombosis No death	None	None	6	3 found on 1 occasion but not subsequently
Marstacimab	26 (8 in cohort 1, 6 in cohort 2, 6 in cohort 3, 7 in cohort 4)	16	No thrombosis No death	None	None	3	None

*TFPI, tissue factor pathway inhibitor; HA, haemophilia A; HAwI, haemophilia A with inhibitors; HBwI, haemophilia B with inhibitors*.

## Befovacimab

Befovacimab, previously known as BAY 1093884, is an IgG2 fully human monoclonal antibody engineered to bind to both the Kunitz 1 (K1) and Kunitz 2 (K2) domains of the TFPI (16). In the preclinical *in vivo* studies, befovacimab showed a pro-coagulant effect, reducing blood loss in mouse and non-human primate bleed models. These were associated with the absence of thrombosis (17). The pharmacokinetic (PK) studies demonstrated bioavailability and a target mediated drug disposition (TMDD) when befovacimab was administered subcutaneously across several *in vivo* models (17).

### Befovacimab Phase 1 Study

In the first-in-human studies, befovacimab was evaluated in a multicentre, open-label study in patients with severe HA or HB, with or without inhibitors (18). Safety, pharmacokinetics (PK) and pharmacodynamics (PD) were assessed after a single intravenous (IV) (0.3 and 1 mg/kg) and single subcutaneous (SC) (1, 3, and 6 mg/kg) dose was given. Total and free TFPI-related parameters showed dose-dependent effects and pharmacokinetics consistent with target mediated drug disposition. The PK and PD were similar to haemophilia A or B with or without inhibitors. In both single-dose and multidose exposure, no safety signals were detected.

### Befovacimab Phase 2 Study

The phase II befovacimab study (Clinicaltrial.org identifier NCT03597022) was a multiple-dose, dose-escalating study evaluating the safety of befovacimab in patients with haemophilia A or B, with or without inhibitors, in three dose cohorts (100, 225, and 400 mg) (19). Eight patients were enrolled in each dose cohort, with 24 study participants in the study. In the study, befovacimab demonstrated a clear pharmacodynamics (PD) effect, with improved protection from bleeds in a dose-dependent manner. Preliminary efficacy analysis revealed a reduction in the bleed rates in the intermediate dose of cohort of 225 mg on befovacimab compared to pre-study bleed rates. The 100 mg dose cohort showed no reduction in bleed rates.

Three participants developed thromboses which were all in the central nervous system. Two of the thromboses were in the intermediate dose cohort (one venous and one arterial) and one in the high dose cohort (1 arterial event). None of the participants was having a bleeding event at the time of thrombosis development, and none received additional haemostatic agents. There was no evidence of haemolysis, platelet consumption, and other monitored laboratory parameters within normal limits. Further analysis revealed no correlation between the befovacimab dose, TFPI inhibition, efficacy and safety outcomes. The absence of specific laboratory findings or any differentiating PK/PD characteristics in patients experiencing serious adverse events raised concerns on the predictability of thrombosis during treatment and led to the sponsor's termination of the study.

## Concizumab

Concizumab, previously called mAb 2021, is an IgG4 humanised monoclonal antibody that binds the K2 domain of the TFPI selectively. *In vitro*, concizumab demonstrated an affinity for both soluble and cell surface-bound TFPI (20). Restoration of thrombin generation was demonstrated *ex vivo* by a dose-dependent reduction in clotting time when concizumab was added to haemophilia blood samples. *Ex vivo* Administration of concizumab significantly reduced cuticle bleeding in haemophilia rabbits when the anti-TFPI antibody was administered 30 min before induction of bleeding. Blood loss was also reduced when administered within 5 min of onset of bleeding (21). These proof of concept data allowed the progression of befovacimab in human clinical trials.

### Concizumab Phase 1 Study

Explorer1 was the first in human dose, phase 1, multicentre, randomised, double-blind, placebo-controlled trial, escalating single intravenous dose (0.5–9,000 μg/kg) or subcutaneous dose (50–3,000 μg/kg) were administered to 28 healthy volunteers or 24 people with haemophilia (22). When given intravenously or subcutaneously, there were no serious adverse events reported. Antidrug antibodies were screened and not seen, and no clinically relevant changes in platelet count, prothrombin time and activated thromboplastin time were reported. The non-linear pharmacokinetics were consistent with target mediated drug disposition.

Explorer3 (NCT02490787) was a multinational, multicentre, randomised, double-blind, placebo-controlled, multiple-dose, dose-escalation, phase 1b trial, conducted at 18 sites in 13 countries (23). It evaluated the safety, pharmacokinetics and pharmacodynamics of concizumab in people with haemophilia. In a 42-day study period, 24 people with haemophilia were randomised in a 3:1 ratio to concizumab or placebo. No serious adverse events and no antidrug antibodies were observed. The PK/PD showed non-linear, dose-dependent pharmacokinetics of concizumab consistent with the target mediated drug disposition. Although the D-dimers and Prothrombin F1+2 were raised at the highest dose used, there were no thrombotic events reported.

### Concizumab Phase 3 Studies

Two phase 2 studies were conducted to evaluate the safety of concizumab in haemophilia patients. Explorer 4 was a multicentre, randomised, open-label, controlled trial evaluating the efficacy and safety of prophylactic administration of concizumab in haemophilia A and B patients with inhibitors (NCT03196284), and Explorer 5 was conducted in haemophilia patients without inhibitors (NCT03196297) (24). The 53 patients enrolled in the two studies comprised of 36 haemophilia patients without inhibitors and 17 patients with inhibitors to FVIII (9 patients) and FIX (8 patients) who received a daily subcutaneous concizumab dose of 15 mg/kg escalated up to 0.25 mg/kg if three or more bleeding events. The estimated annualised bleed rate in haemophilia A (HA) with inhibitors (HAwI), haemophilia B with inhibitors (HBwI) and HA without inhibitors were 3.0, 5.9, and 7, respectively. Most (88%) of inhibitor patients did no escalate the dose. There were 21 (58.3%) inhibitor and non-inhbitor patients whose concizumab dose was escalated (58.3%) of which 7 (19.4%) were escalated to 0.2 mg/kg and 8 (22.2%) were escalated to 0.25 mg/kg. Three patients developed low titre antidrug antibodies in each of the trials, and these antibodies did not have a clinical effect. Overall, patients on concizumab had lower ABRs when compared to eptacog alfa.

### Concizumab Phase 3 Study

The acceptable efficacy and safety profile from phase 2 studies led Novo Nordisk to initiate the explorer7 phase 3 clinical trial in October 2019 with concizumab in patients with haemophilia A or B with and without inhibitors. A parallel phase 3 trial in haemophilia A or B patients without inhibitors, explorer8, was initiated in November 2019. The two trials' objective was to establish the safety and efficacy of once-daily prophylactic subcutaneous concizumab delivered in a pen device to prevent bleeds. The recruitment target was 293 patients from 32 countries. The trials were paused in March 2020 due to non-fatal thrombotic events in three patients enrolled in the ongoing phase 3 programme. Novo Nordisk worked with relevant regulatory authorities and identified a new path forward for concizumab. When new mitigating safety measures and guidelines were put in place, the study hold was lifted, and there has been no new incidence since measures were in place.

## Marstacimab

Marstacimab, previously known as PF-06741086, is a fully human monoclonal antibody IgG1 antibody that binds to the K2 domain of TFPI at nanomolar to suprananomolar concentration ranges (25). Non-clinical experiments demonstrated that marstacimab could enhance thrombin generation *in vitro* and *in vivo* in a concentration-dependent manner (26). *In vitro* thrombin generation assays (TGAs) and dilute prothrombin time (dPT) assays evaluating Marstacimab added to donor plasma from healthy normal volunteers and patients with haemophilia A, haemophilia B, and haemophilia A with inhibitors have all shown the ability of marstacimab to increase coagulation activity and promote haemostasis. Bleed control was demonstrated in severe injury mice models of haemophilia A and B (25, 26).

### Marstacimab Phase 1 Study

A first-in-human study of the safety, tolerability, pharmacokinetics and pharmacodynamics of marstacimab was conducted in 42 healthy male volunteers (27). They received a dose range from 30 to 440 mg intravenously and subcutaneously. Volunteers receiving low dose were followed up for 42 days, and dose assigned to the high dose cohort were followed up for 84 days. A single dose of marstacimab at multiple dose levels was safe and well-tolerated in a healthy adult male population. The safety, PK and PD data from this study supported marstacimab development to a multiple-dose study in haemophilic patients.

### Marstacimab Phase 1b/2 Study

This study (clinicatrial.gov identifier NCT02531815) investigated the safety, efficacy, pharmacokinetics, and pharmacodynamics of marstacimab, in haemophilia A or B patients, with or without inhibitors. This was a multicentre study conducted at ten sites in three countries. Twenty-six participants were exposed to Marstacimab in four dose cohorts (300 mg non-inhibitor, 150, 450, and 300 mg inhibitor cohorts) (28). The post-dose follow-up was for 113 days. Three participants developed antidrug antibodies, none of which had a neutralising effect. There were four severe adverse events, none of which were related to marstacimab. No thrombotic AEs were observed, no clinically significant changes in coagulation laboratory parameters were reported in this study.

Preliminary efficacy was evaluated by comparing the study participant bleed rates in the 6 months before the study and then 6 months on study. The ABR was zero for most of the dose cohorts, and the reduction in the bleed rate was 80–96% across the four-dose cohorts. These data supported the progression of this study to phase 3 of marstacimab clinical development.

## MG1113

MG1113 is humanised IgG4 monoclonal antibody that binds to the Kunitz-2 domain (K2) of TFPI. It has been shown *in vitro* to restore thrombin generation in animals deficient in FVIII or FIX with and without inhibitors (29). In the *ex vivo* experiments, spiking haemophilia plasmas with MG1113 increased thrombin generation in a concentration-dependent fashion. *In vivo*, intravenous and subcutaneous injection of MG1113 resulted in a reduction in blood loss. In the pharmacokinetic and pharmacodynamics evaluation, MG1113 showed a non-linear PK after both IV and SC administrations at the dosing range from 2.5 to 10 mg/kg (30). This is consistent with the target mediated drug disposition which has been demonstrated with all anti-TFPI monoclonal therapeutics.

## Conclusion

The phase 1 and phase 2 data reviewed indicates that progress in developing anti-TFPI therapies is promising, with two of these molecules progressing to phase 3 evaluation. The occurrence of thrombotic events should remind us that the safety of these novel therapies should come first, and the drugs should be thoroughly evaluated before they are accepted as a standard of care. Recent experience with development of FVIII mimetics indicate that thrombotic events can be mitigated by better pharmacokinetic understanding and appropriate dose adjustment.This gives optimism that an anti-TFPI with an acceptable profile may be identified in the currently ongoing clinical studies. If the currently evolving safety and efficacy data remain positive and acceptable; the anti-TFPI therapies may represent a significant advance in the care of patients with haemophilia A or B with and without inhibitors.

## Author Contributions

JM was responsible for the conception, content research, data collection, analysis, and approval of this manuscript's final version.

## Conflict of Interest

The author declares that the research was conducted in the absence of any commercial or financial relationships that could be construed as a potential conflict of interest.
